# Influence of a Cardiac Rehabilitation Program on Renal Function in Patients With Cardiovascular Disease in a One-Year Follow-Up

**DOI:** 10.14740/cr440e

**Published:** 2015-10-25

**Authors:** Kanta Fujimi, Shin-ichiro Miura, Takuro Matsuda, Masaomi Fujita, Yoshiyuki Ura, Kouji Kaino, Maya Sakamoto, Tomoe Horita, Tadaaki Arimura, Yuhei Shiga, Keijiro Saku

**Affiliations:** a Department of Cardiology, Fukuoka University School of Medicine, Fukuoka, Japan; b Department of Molecular Cardiovascular Therapeutics, Fukuoka University School of Medicine, Fukuoka, Japan; c Department of Rehabilitation, Fukuoka University Hospital, Fukuoka, Japan; d Division of Nutrition, Fukuoka University Hospital, Fukuoka, Japan

**Keywords:** Exercise training, Cardiac rehabilitation, Estimated glomerular filtration rate, Renal function

## Abstract

**Background:**

Exercise training may improve renal function in patients with chronic kidney disease (CKD). The effect of cardiac rehabilitation (CR) with exercise training on renal function has not yet been established. We evaluated the effects of CR on renal function in patients with cardiovascular disease (CVD).

**Methods:**

Twenty-three CVD patients in a 1-year CR program (CR group) who had ischemic heart disease (IHD) and/or heart failure were compared with 26 age- and gender-matched CVD patients without CR (non-CR group, standard pharmacological care alone). At baseline and after 1 year, urea nitrogen (UN), creatinine (Cr), potassium (K), estimated glomerular filtration rate (eGFR) and hematocrit (Hct) in blood were assessed.

**Results:**

There were no differences in the patient characteristics at baseline between the CR and non-CR groups except for the percentages of heart failure and the use of calcium channel blocker. After 1 year, there were no significant changes in UN, Cr, K, eGFR or Hct in either the CR or non-CR groups. The patients in the CR group were divided into two groups according to the eGFR level at baseline: low (n = 12, eGFR < 51 mL/minute/1.73 m^2^, indicating mild-to-moderate CKD) and high (n = 11, eGFR ≥ 51 mL/minute/1.73 m^2^) eGFR groups. Although there were no differences in the patient characteristics at baseline between the low and high eGFR groups, the low eGFR group showed a significant increase in eGFR after the 1-year CR program.

**Conclusions:**

CR may improve renal function in patients with mild-to-moderate CKD.

## Introduction

Cardiac rehabilitation (CR) with exercise training has been shown to improve exercise capacity and prognosis in patients with cardiovascular disease (CVD) [1].

Chronic kidney disease (CKD) is a severe health-related problem [2]. Exercise training may deteriorate renal function [3], because exercise may cause a transient fall in renal blood flow [4]. In addition, previous studies have shown that exercise training in patients with CKD was not associated with an improved estimated glomerular filtration rate (eGFR) [5, 6]. On the other hand, Pechter et al reported that exercise training significantly diminished proteinuria and cystatin C and enhanced GFR in CKD patients [7]. Other studies also demonstrated that exercise training improved renal function in CKD patients with or without CVD [8, 9]. Thus, the effect of exercise training on renal function remains controversial. In addition, it remains unclear whether CR with exercise training improves renal function in patients with CVD.

Accordingly, the aim of this study was to determine whether CR with exercise training is associated with the amelioration or deterioration of renal function in patients with CVD.

## Methods

### Study population and protocol

Twenty-three CVD patients who had ischemic heart disease (IHD) and/or heart failure (HF) and participated in a CR program (CR group) were retrospectively enrolled. In addition, 26 age- and gender-matched CVD patients without CR (non-CR group) were also selected. All data were collected at baseline and after 1 year. The study was approved by the ethics committee of Fukuoka University Hospital. We retrospectively collected and analyzed all data using the database of Fukuoka University Hospital.

### Exercise protocol

The CR group participated in a supervised exercise training program at the hospital’s gym 2 - 3 times 1 week for 1 year. Exercise intensity was chosen at the estimated 50% of peak VO_2_ according to heart rate and Borg’s scale 11-13 during exercise as referenced by previous reports [10, 11]. Each session lasted about 1 h, beginning with a warm-up exercise for 10 min, followed by 30 min of cycling or walking at the indicated exercise intensity and 20 min of cooling down and stretching. Blood pressure and heart rate were measured at rest and at the end of exercise, and an electrocardiogram (Central Monitor (DS-5700), Fukuda Denshi Co., Ltd, Tokyo, Japan) and Borg’s scale were recorded during exercise. All patients were routinely screened before each exercise session, such as by symptoms, heart rate and rhythm, electrocardiogram, blood pressure and medication regimen. The following conditions had to be managed during exercise: angina, dysrhythmia, hypotension, hypertension, dyspnea, decreased exercise tolerance and cardiac or respiratory arrest. Emergency equipment was immediately available in the exercise area, and the emergency cart, resuscitation equipment and medications were checked regularly. Our CR staff is well trained at administering basic life support and advanced cardiac life support.

### Data collection

Fasting blood samples were collected at baseline and after 1 year. Patient characteristics, including medications at baseline and urea nitrogen (UN), creatinine (Cr), eGFR, potassium (K) and hematocrit (Hct) in blood, were assessed at baseline and after 1 year.

Patients with low-density lipoprotein cholesterol ≥ 140 mg/dL, triglyceride ≥ 150 mg/dL or high-density lipoprotein cholesterol < 40 mg/dL and lipid-lowering therapy were diagnosed with dyslipidemia (DL). Patients with systolic or diastolic blood pressure ≥ 140 mm Hg or 90 mm Hg or who were under antihypertensive treatment were considered to have hypertension (HTN). Patients who were being treated for diabetes mellitus (DM) or who had symptoms of DM and a fasting glucose concentration ≥ 126 mg/dL were considered to have DM. Otherwise, the results of a 75 g oral glucose tolerance test were used to diagnose DM. IHD was defined as lumen diameter stenosis > 50% in at least one major coronary artery as determined by coronary angiography and as diagnosed by old myocardial infarction. HF was assumed based on the medical history including medications and cardiac function.

### Statistics

Statistical analysis was performed using the Stat View statistical software package (Stat View 5; SAS Institute INC., Cary, NC). Data are expressed as the mean ± standard deviation or number (%). The significance of differences was evaluated using Wilcoxon signed-rank test or Student’s *t*-test for continuous variables and the χ^2^ test for categorical variables. A value of P < 0.05 was considered significant.

## Results

### Patient characteristics at baseline in the CR and non-CR groups

The patient characteristics at baseline are presented in Table 1. In the CR group, the mean age was 69 ± 10 years and the percentage (%) of males was 82%. In addition, %HTN, %DM, %DL, %HF and %IHD were 87%, 30%, 65%, 83% and 61%, respectively. In the non-CR group, the mean age, % male, %HTN, %DM, %DL, %HF and %IHD were 69 ± 11 years, 58%, 70%, 23%, 70%, 27% and 58%, respectively. There were no differences in any of the baseline characteristics except for %HF and % calcium channel blocker (CCB) between the CR and non-CR groups. The CR group showed significantly higher %HF and lower %CCB than the non-CR group.

**Table 1:** Patient Characteristics at Baseline in the Non-CR and CR Groups

	Non-CR group(n = 26)	CR group (n = 23)
Age, years	69 ± 11	69 ± 10
Male, n (%)	15 (58)	19 (82)
HTN, n (%)	18 (70)	20 (87)
DM, n (%)	6 (23)	7 (30)
DL, n (%)	18 (70)	15 (65)
HF, n (%)	7 (27)	19 (83)*
IHD, n(%)	15 (58)	14 (61)
Medication		
ARB/ACE-I, n (%)	14 (54)	13 (57)
Statin, n (%)	13 (50)	9 (39)
Diuretics, n (%)	6 (23)	11 (48)
CCB, n (%)	19 (73)	11 (48)*
β-blocker, n (%)	18 (69)	13 (57)

HTN: hypertension; DM: diabetes mellitus; DL: dyslipidemia; HF: heart failure; IHD: ischemic heart disease; ARB: angiotensin II receptor blocker; ACE-I: angiotensin converting enzyme inhibitor; CCB: calcium channel blocker. *P < 0.05 vs. non-CR group.

### Effects of CR on renal function, K and Hct in blood

Next, we assessed changes in renal function between baseline and after 1 year in the CR and non-CR groups (Table 2). There were significant differences in Cr and Hct at baseline between the two groups. Neither group showed significant changes in UN, Cr or eGFR, or in K or Hct in blood between baseline and after 1 year.

**Table 2:** Biochemical Parameters in Blood at Baseline and at 1-Year in the Non-CR and CR Groups

	Non-CR group (n = 26)		CR group (n = 23)	
	Baseline	1 year	Baseline	1 year
BUN (mg/dL)	16 ± 4.4	16 ± 5.1	19 ± 6.2	18 ± 4.4
Cr (mg/dL)	0.9 ± 0.2	0.9 ± 0.1	1.1 ± 0.3*	1.1 ± 0.2
eGFR (mL/min)	60 ± 12	60 ± 12	54 ± 14	53 ± 11
K (mmol/L)	4.5 ± 0.6	4.4 ± 0.4	4.4 ± 0.5	4.4 ± 0.6
Hct (%)	42 ± 4.2	43 ± 4	37 ± 8.7*	40 ± 3.6

UN: urea nitrogen; Cr: creatinine; eGFR: estimated glomerular filtration rate; K: potassium; Hct: hematocrit. *P < 0.05 vs. at baseline in the non-CR group.

### Baseline characteristics in patients with low and high eGFR in the CR group

Since there were no changes in UN, Cr or eGFR between baseline and after 1 year in the CR group, the patients in the CR group were divided into two groups according to the eGFR level at baseline: low (n = 12, eGFR < 51 mL/min/1.73 m^2^) and high (n = 11, eGFR ≥ 51 mL/min/1.73 m^2^) eGFR groups (Table 3). There were no differences in any of the patient characteristics at baseline, including medications, between the low and high eGFR groups.

**Table 3:** Patient Characteristics at Baseline in the High and Low eGFR Groups

	High eGFR group (n = 11)	Low eGFR group (n = 12)
Age, years	67 ± 12	72 ± 9
Male, n (%)	8 (72)	10 (83)
HTN, n (%)	10 (91)	10 (83)
DM, n (%)	2 (18)	5 (42)
DL, n (%)	7 (64)	8 (67)
HF, n (%)	8 (72)	11 (92)
IHD, n (%)	8 (72)	6 (50)
Medication		
ARB/ACE-I, n (%)	6 (55)	7 (58)
Statin, n (%)	6 (55)	3 (25)
Diuretics, n (%)	3 (27)	8 (67)
CCB, n (%)	4 (36)	7 (58)
β-blocker, n (%)	6 (55)	7 (58)

HTN: hypertension; DM: diabetes mellitus; DL: dyslipidemia; HF: heart failure; IHD: ischemic heart disease; ARB: angiotensin II receptor blocker; ACE-I: angiotensin converting enzyme inhibitor; CCB: calcium channel blocker.

### Effects of CR on renal function in patients with low and high eGFR in the CR group

**Figure 1:**
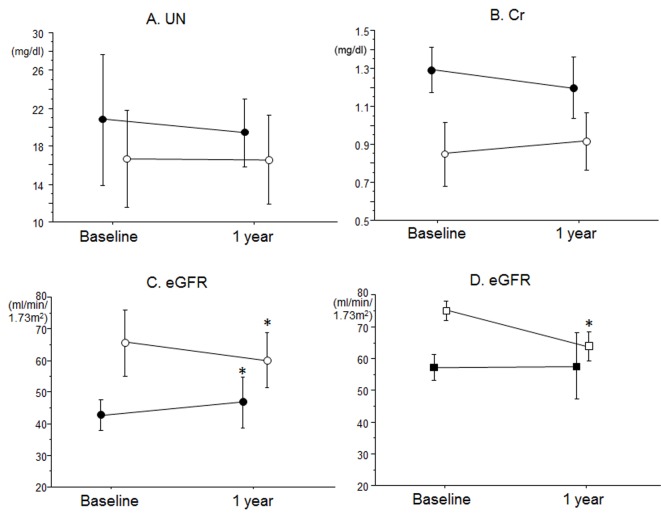
Effects of CR on (A) UN, (B) Cr and (C) eGFR in blood in patients with low eGFR (closed circles) and high eGFR (open circles) in the CR group. (D) Effects of CR on eGFR in blood in patients with eGFR ≥ 70 (open squares) and < 70 mL/min/1.73 m^2^ (closed squares) at baseline in the high eGFR group. *P < 0.05 vs. at baseline.

Finally, we analyzed changes in renal function between baseline and after 12 months in patients with low and high eGFR in the CR group (Fig. 1). There were no differences in UN or Cr between the groups (Fig. 1A, B). Interestingly, the low eGFR group showed a significant increase in eGFR after a 1-year CR program, whereas the high eGFR group showed a significant decrease in the eGFR level (Fig. 1C). In addition, patients with high eGFR in the CR group were divided into two groups according to the eGFR level at baseline: eGFR ≥ 70 mL/min/1.73 m^2^ (n = 6) and eGFR < 70 mL/min/1.73 m^2^ (n = 5) groups (Fig. 1D). The eGFR at baseline (75 ± 3 mL/min/1.73 m^2^) in the eGFR ≥ 70 mL/min/1.73 m^2^ group significantly decreased after 1 year (56 ± 4 mL/min/1.73 m^2^). On the other hand, there were no significant changes in eGFR between baseline (56 ± 4 mL/min/1.73 m^2^) and after 12 months (55 ± 10 mL/min/1.73 m^2^) in the eGFR < 70 mL/min/1.73 m^2^ group.

## Discussion

The major findings of the present study are: 1) overall, eGFR remained unchanged in both the CR and non-CR groups; and 2) renal function improved in patients with low eGFR, but not high eGFR, in the CR group.

The mechanisms of the improvement in renal function with exercise training are not yet clear. Generally, exercise training decreases renal blood flow and GFR [12, 13]. One proposed mechanism is that exercise training reduces excessive sympathetic nerve activity [14], since the progression of renal disease is affected by sympathetic nerve activity. Exercise training may improve the GFR via an attenuation of excessive sympathetic nerve activity. In the present study, since renal function improved in patients with low eGFR in the CR group, these mechanisms should be considered.

It remains unclear whether the effects of exercise training on renal function differ according to the severity of renal function. The improvement in eGFR after CR was observed only in patients with mild-to-moderate CKD, but not in those with severe CKD [9]. One possible explanation is that functional renal reserve capacity is irreversibly deteriorated in severe CKD [15]. In this study, the low eGFR group included mild-to-moderate CKD (43 ± 5.3 mL/min/1.73 m^2^), but not severe CKD. Therefore, we may observe improved renal function in patients with mild-to-moderate CKD in the CR group. On the other hand, the high eGFR group showed a significant decrease in the eGFR level in this study. Since the change in the level of Cr between baseline and after 1 year was within the normal range in the high eGFR group, and since exercise training generally induces an increase in serum Cr levels, there may be no problem with changes in eGFR in the high eGFR group. In addition, the patients with eGFR ≥ 70 mL/min/1.73 m^2^ at baseline in the high eGFR group, but not those with eGFR < 70 mL/min/1.73 m^2^, showed a significant decrease after 1 year. These patients may be in a state of glomerular hyperfiltration, which is a functional abnormality of the kidney, and CR might help them to recover from this condition. We need to confirm this observation by analyzing changes in the levels of urinary albumin and serum cystatin C after a 1-year CR program.

A favorable exercise intensity is also important for improving renal function. A high intensity at 60-75% of heart rate reserve in patients with moderate-to-advanced CKD did not change renal function [3, 6]. Exercise at a moderate intensity (50-60% of heart rate reserve or Borg’s scale 12-13) had a favorable effect on renal function in patients with mild-to-moderate CKD [7, 8]. The present study showed that exercise at a moderate intensity (50% of peak VO_2_ according to heart rate reserve and Borg’s scale 11-13) had a beneficial effect on renal function.

The patients in the CR group had either HF, IHD or both. CKD is an important risk factor for IHD. In addition, in addition to renal dysfunction, anemia is closely associated with the progression of HF [16]. There was no change in Hct between baseline and 1 year in the CR group. CR is known to improve coronary risk factors and prognosis in patients with IHD [1] and cardiac function in HF patients [11]. Therefore, the present study provides evidence that patients with IHD and/or HF complicated with mild-to-moderate renal dysfunction may benefit from CR.

There are several limitations to the present study. First, it was retrospective and from a single center with a relatively small sample size. Second, we measured renal function only at baseline and after 1 year. Third, we did not evaluate urinary albumin levels. Therefore, a large controlled randomized study should be performed to confirm that CR improves renal function.

In conclusion, CR may improve renal function in patients with mild-to-moderate CKD.
